# Sex differences in expression of CGRP family of receptors and ligands in the rat trigeminal system

**DOI:** 10.1186/s10194-024-01893-1

**Published:** 2024-11-08

**Authors:** Aida Maddahi, Jacob C. A. Edvinsson, Lars Edvinsson

**Affiliations:** Department of Clinical Sciences, Division of Experimental Vascular Research, Lund University, Lund, Sweden

**Keywords:** Trigeminal ganglion, CGRP, Adrenomedullin, Amylin, Calcitonin, Receptors, Migraine

## Abstract

**Background:**

Calcitonin gene-related peptide (CGRP) is part of the calcitonin peptide family, which includes calcitonin (CT), amylin (AMY), and adrenomedullin (ADM). CGRP and its receptor are highly present in the trigeminovascular system (TVS). Recent research suggests that other members of the calcitonin family could be feasible therapeutic targets in the treatment of migraine. The present study aims to elucidate the distribution of ADM, AMY, CT, and their receptors in the rat TVS, and to explore potential sex differences in their expression.

**Methods:**

Trigeminal ganglia (TG) were dissected from male and female adult rats. Protein and gene expression were assessed through immunohistochemistry and RT-qPCR. Additionally, the dura mater was isolated for further investigation of protein expression and fiber localization using immunohistochemistry.

**Results:**

Quantitative gene expression analysis revealed the presence of all genes in male and female TGs, except for calcitonin receptor (CTR). Notably, CGRP mRNA levels in TG were several folds higher than those of other genes. The receptor activity-modifying protein-1 (RAMP1) mRNA levels were significantly higher in female compared to male.

No AMY or CT immunoreactivity was observed in the TVS. In contrast, immunoreactivity for ADM, CGRP, RAMP1, CTR, and calcitonin-like receptor (CLR) were observed in the cytoplasm of TG neurons. Immunoreactive Aδ-fibers storing RAMP1, ADM and CLR were also identified. RAMP2 and RAMP3 were expressed in nucleus of TG neurons and in satellite glial cells. Furthermore, RAMP1 and CLR were co-localized with CASPR in the nodes of Ranvier located in Aδ-fibers.

**Conclusions:**

This study provides valuable insights into the distribution of the CGRP family of peptides and their receptors in the TVS. CGRP mRNA levels in the TG were markedly higher than those of other genes, demonstrating the key role of CGRP. The co-localization of CLR and RAMP1 on Aδ-fibers with CASPR suggests a potential role for this receptor in modulating trigeminal nerve function and neuronal excitability, with implications for migraine pathophysiology. Additionally, RAMP1 mRNA levels were significantly higher in female TG compared to males, indicating sex-specific differences in gene expression. These findings underscore the need for further research into the functional significance of gender-related variations.

**Supplementary Information:**

The online version contains supplementary material available at 10.1186/s10194-024-01893-1.

## Introduction

Migraine is a prevalent primary headache disorder affecting up to 15% of the world’s population [1, 2]. Migraine ranks among the most burdensome disorders and has enormous personal and socioeconomic consequences and, is number one in the world’s case disability among female’s in their most active ages 15–49 years [3]. Women are disproportionally affected by migraine, with up to three time’s higher prevalence than men [4, 5]. Women also report a longer attack duration, increased risk of headache recurrence, greater disability, and a longer period of time required to recover [6, 7]. The dynamic regulation of female sex hormones during the menstruation cycle has been proposed to influence the high prevalence of migraine in females [8].

Calcitonin gene-related peptide (CGRP) is a 37 amino acid neuropeptide that is widely expressed in both central and peripheral nervous systems [9]. As a neuromodulator, CGRP has been shown to have a key role in the pathophysiology of migraine pain through sensitization of the trigeminovascular system (TVS) [10, 11]. CGRP and its receptors are richly expressed in the trigeminal ganglion (TG) neurons [12]. The neuron cell bodies in the TG can be categorized into two main groups: small to medium sized neurons (∼30–60 μm) that store CGRP and project unmyelinated C-fibers, and large sized (> 60 μm) neurons that contain CGRP receptors and project lightly myelinated Aδ-fibers [12].

CGRP belongs to the calcitonin peptide family that in addition includes calcitonin (CT), amylin (AMY), and adrenomedullin (ADM). CGRP itself exists in two forms, αCGRP and βCGRP which share almost 90% structural similarity with only three amino acid differences [13, 14]. In addition, αCGRP has been described to be the predominant form in the central and peripheral nervous system while βCGRP is more relatively abundant in the enteric nervous system [14].

The calcitonin family of peptides has a wide range of biological and physiological functions, including regulation of calcium homeostasis, vascular tension, inflammation, feeding behavior and pain recognition [14–16]. CGRP is a multifunctional neuropeptide which is known to regulate the cardiovascular system, mediate neurogenic inflammation, and modulate nociceptive input [16]. So far, CGRP is the outstanding member of the calcitonin family of neuropeptides that is associated with migraine [17]. However, expanding evidence propose that other members of this family including the peptides amylin and adrenomedullin may also play a role [18, 19].

Receptors for the calcitonin peptide family are complex and consist of two class B G protein-coupled receptors (GPCRs), the calcitonin receptor (CTR) and the calcitonin-like receptor (CLR). Both CLR and CTR can interact with accessory proteins known as receptor activity-modifying proteins (RAMPs) [18, 20]. This results in the CGRP receptor (CLR/RAMP1), the ADM1 (CLR/RAMP2), and the ADM2 (CLR/RAMP3) receptors. Other known receptor complexes are the AMY1 (CTR/RAMP1), AMY2 (CTR/RAMP2), and AMY3 (CTR/RAMP3) receptors. CLR is unable to translocate to the cell surface or act as a peptide-ligand receptor on its own. In contrary, CTR can translocate to the cell surface and is able to act as a receptor by itself. Further, CGRP has been shown to activate both CLR/RAMP1 and CTR/RAMP1 [21, 22].

Despite the enormous burden of migraine among women relative to men, mechanisms behind the sex disparities are understudied [23]. Recent works have examined the distribution of sex related hormones (estrogen and progesterone) and hypothalamic hormones (oxytocin and vasopressin), and their receptors, within the brain and in the TVS [8]. So far, we have demonstrated difference in the expression of immunoreactive neurons between males and females in TG for the estrogen receptors (ERα, ERβ, and GPER), progesterone receptor-A (PR-A), vasopressin receptor 1a (V1aR), oxytocin (OT), oxytocin receptor (OTR) and the CGRP receptor component RAMP1, where female TG, compared to male, contained more immunoreactive neurons [24–28].

In the present study we aimed to explore whether sex differences exist in the expression and distribution of the CGRP family of peptides and receptors within the TVS, potentially elucidating the sex disparities observed in migraine prevalence. For this purpose, we used quantitative mRNA analysis using RT-qPCR, and protein expression was explored using immunohistochemistry. In addition, the expression of CGRP family receptors was explored in rat dura mater to determine whether peripheral extrasynaptic signaling could be relevant for amylin and adrenomedullin.

The suggestion of sex-specific sensitivities to peptides within the CGRP family adds complexity to understanding migraine pathophysiology. The structural and receptor cross-reactivity shared by CGRP, CT, AMY, and ADM underscores their collective biological impact [29].

## Experimental procedures

### Animals

All animal procedures in this study followed the guidelines of the European Communities Council (86/609/ECC) and were approved by the Regional Ethical Committee on Animal Research, at the University of Lund, Sweden (LU-818-01). Animals were housed under controlled temperature and humidity with free access to water and food.

### Tissue preparation

A total of 24 adult Wistar rats were divided in 2 groups (12 males and 12 females, weights ranging between 250 and 300 g) and were used for immunohistochemistry and RT-qPCR. The rats were anaesthetized with CO_2_ and subsequently decapitated. The undamaged regions of dura mater (inside the temporal area of the skull from only male rats), basilar artery and TGs (left and right from both male and female rats), were carefully dissected. The dura mater segments were after a wash in phosphate buffer saline containing 0.3% Triton X-100 (PBST), spread out on microscope slides (Super frost. ThermoFisher) and allowed to dry. Thereafter the dura mater, left and right TG and basilar arteries were fixed in 4% paraformaldehyde (PF) in phosphate buffer saline (PBS) for 3–4 h at room temperature for immunohistochemistry or immediately fresh frozen in liquid nitrogen for RT-qPCR (only TGs). For immunohistochemistry, after fixation the tissues were incubated overnight in Sörensen’s phosphate buffer (pH 7.2), gradient containing 10% and 25% sucrose for cryoprotection. Then, the microscope slides with dura mater spreads were dried of excess fluid and kept at -20 °C. The TGs and basilar arteries were embedded in an egg albumin-based protein medium and sectioned at a thickness of 10 μm with a cryostat (Microm Cryo Star HM 560). Finally, the sections were collected on microscope slides and stored at -20 °C until use.

### Total RNA extraction and RT-qPCR

The TG from six male and six female rats were carefully dissected. For each rat, the left and right TG were pooled together as a single sample and collected in 2 ml Eppendorf tubes (Safe –lock tubes 2 ml, Eppendorf AG, 22331 Hamburg, Germany). The samples were immediately frozen in liquid nitrogen for RNA extraction. All RNA extraction was performed using RNeasy^®^ Plus Mini kit (Qiagen, Hilden, Germany) according to the manufacturer’s protocol. Concentration of the total RNA was determined using a GeneQuant Pro spectrophotometer (Amersham Pharmacia Biotech, Uppsala, Sweden). A ratio of sample absorbance at 260 /280 nm in the range of 1.8 to 2 was acceptable and indicating a high RNA purity. First-strand cDNA was prepared from 1 µg of total RNA in a 20 µL reverse transcript reaction using Superscript^®^ III First-Strand Synthesis Super Mix (Invitrogen, Carlsbad, CA, USA). A reverse transcription negative control for each sample to detect the genomic DNA was performed simultaneously and underwent the same procedures but without Superscript III Reverse Transcriptase (RT enzyme). The cDNA obtained was diluted four times and stored at -20 °C. The sequences of primers for RT-qPCR were specific for the genes of interest and were listed in Table 1. The housekeeping gene glyceraldehyde-3-phosphate dehydrogenase (GAPDH) was used as a reference gene which the gene expressions were normalized against.

**Table 1:** List of primer ssequences used for RT-qPCR

Gene name	Forward primer Sequence (5´- 3´)	Reverse primer Sequence (5´- 3´)
Rat CGRP	TGTGTCAGAAAGGCTGATGG	CCGCTTGAGGTTTAGCAGAG
Rat AMY	CTGCCAGCTGTTCTCCTCAT	TGGAGCGAACCAAGAAGTTT
Rat ADM	GTGGAATAAGTGGGCGCTAA	ATGCCGTCCTTGTCTTTGTC
Rat CLR	CCAACGGATTACATTGCATA	CAGTAAAGCAGCACAAATGG
Rat CTR	ATGAGGTTCCTTCTCCTGAACAGG	CGGTCATAGCACTTGTACTGAGCA
Rat RAMP1	GAGGACATGGAGACCATAGG	CAGTCATGAGCAGTGTGACC
Rat RAMP2	CTGGGGTATTGCTTGGAGTA	TAACGAGGAAAGGGATGAGG
Rat RAMP3	CTTCTCCCTCTGTTGCTGCT	AGCCCACGATGTTTGTCTCC
Rat GAPDH	CTGCACCACCAACTGCTTAGC	TCAGCTCTGGGATGACCTTGC

The RT-qPCR was performed in 20 µL reaction consisting of 2 µL diluted cDNA, 0.5µM of each primer, 10 µL Fast SYBR™ Green Master Mix (Applied Biosystems, CA, USA), and 7 µL RNase free water in a Step One Plus Real Time PCR System (Applied Biosystems, CA, USA) with the following thermal profile: Holding stage at + 50 °C for 2 min and + 95 °C for 10 min, followed by 40 PCR cycles at + 95 °C for 15 s and + 60 °C for 1 min. Each sample was examined in duplicate, and a blank control (without template) was used in all experiments. After amplification a melting curve analysis was performed to verify that each primer pair generated only one PCR product of the expected size.

### Immunohistochemistry

The TG and basilar artery sections from six male and six female rats, along with dura mater from only six male rats, were washed and permeabilized in PBST for 15 min. Thereafter, the sections were blocked for non-specific binding of antibodies for 1 h in blocking solution containing PBST, 1% bovine serum albumin (BSA), and 5% normal serum (depending on species origin of the secondary antibodies). After blocking, the sections were incubated overnight at + 4 °C in moisturized chambers with primary antibodies. The primary antibodies using in the present study were the same antibodies used in a previous study [30], except for calcitonin and amylin antibodies (for details, see Table 2). The following day, the sections were washed in PBST for 2 × 15 min followed by incubation with secondary antibodies (for details, see Table 3). All antibodies were diluted in PBST containing 1% BSA. The sections were subsequently washed with PBST for 2 × 15 min and mounted with an anti-fading mounting medium containing 4’, 6-diamidino-2-phenylindole (DAPI) (Vectashield, Vector Laboratories, Burlingame CA, USA). Each procedure was repeated three times to validate the results and to minimize any experimental errors. Further, negative controls were included by omitting the primary antibody to evaluate non-specific secondary antibody binding.

For details and specification of primary and secondary antibodies see previous study [30]. In addition, the specificity of CGRP, CLR and RAMP1 antibodies have been evaluated by Western blot and pre-absorption controls experiments, in a previous study at our laboratory [12].

**Table 2:** Primary antibodies are used for immunohistochemistrys

Name and product code	Dilution	Host	Supplier
CGRP(ab81887)	1:100	Mouse	Abcam, Cambridge, UK
Adrenomedullin (Sc-80462)	1:100	Mouse	Santa Cruz Biotechnology, CA, USA
Amylin (SC-377530)	1:100	Mouse	Santa Cruz Biotechnology, CA, USA
Amylin (LS-C352341)	1:200	Rabbit	Life Span Bioscience, WA, USA
Amylin (PA5-32261)	1:100	Rabbit	ThermoFisher Scientific, MA, USA
Calcitonin ( bs-18210R)	1:50	Rabbit	Bioss Inc, Woburn, USA
Calcitonin (ab16697)	1:50	Rabbit	Abcam, Cambridge, UK
CTR (CAU24308)	1:100	Rabbit	BioMatik, Delaware, USA
CLR (3155)	1:500	Rabbit	Merck & Co., Inc., USA
RAMP1(844)	1:100	Goat	Merck & Co., Inc., USA
RAMP2 (GTX108524-S)	1:100	Rabbit	GeneTex, USA
RAMP3 (SC-365313)	1:100	Mouse	Santa Cruz Biotechnology, CA, USA

**Table 3:** Secondary antsibodies are used for immunohistochemistry

Name	Dilution	Against	Supplier
Alexa 488	1:100	Goat	Thermo Scientific, IL, USA
Alexa 549	1:200	Mouse	Thermo Scientific, IL, USA
FITC	1:100	Mouse	Jackson Immunoresearch, West Grove, PA, USA
Alexa 594	1:200	Rabbit	Thermo Scientific, IL, USA
FITC	1:100	Rabbit	Jackson Immunoresearch, West Grove, PA, USA

Double immunohistochemistry was performed using antibodies against ADM, CTR, CLR, RAMP1, RAMP2 and RAMP3 in combination with either CGRP or contactin- associated protein 1 (CASPR). CASPR is an antibody marking the paranodal regions of myelinated axons. All procedures were the same as described above, albeit the primary antibodies were mixed and applied together.

Immunoreactivity of the TG, basilar artery and dura mater was visualized using an epifluorescence microscope (Nikon 80i; Tokyo, Japan) at the appropriate wavelengths and photographed with an attached Nikon DS-2Mv camera. Images were processed using Adobe Photoshop CS3 (v.10.0, Adobe Systems, Mountain View, CA).

### Examinatiosn of AMY/ADM and their receptor elements in rat dura mater

Paracrine signaling between C-fibers and Aδ-fibers is considered a possible route for CGRP signaling in the trigeminal system [14, 31]. Because of this, it is of interest to examine if other members of the CGRP family of receptors also are expressed in a similar manner as RAMP1 and CLR. This would indicate if AMY and ADM could modify the pain signal traveling along the axons. Most of the dura mater consists of connective tissue and blood vessels, which were analyzed in detail, both for fibers and blood vessel walls.

### Cell counting

Cell counting was performed to semi-quantify expression of CGRP peptides family and their receptors in TG. Three slides with three sections between each were used for measurements. Cell counting was performed in the central area of the TGs. Due to the risk of artefactual florescence, cell counting was avoided close to where the TG bordered the surrounding embedding medium. Images were taken of the representative area (0.75 mm^2^) at 10 x magnification. A NIS-elements BR image analysis program (Nikon) was used to calculate the number of cells and to measure the fluorescence intensity in each area. All cells in this area, including negative and positive cells, were counted. The mean percentage of positive neurons in 3 slides (contained 3 sections per each slide)/rat, from all rats (*n* = 6) was used for analysis. The intensity measurements were used to verify that immune-positive cells were correctly distinguished from negative cells.

### Calculations and statistical analysis

Real-time PCR data were analyzed with comparative cycle threshold (C_t_) method [32]. The C_t_ values of GAPDH, CGRP, ADM, AMY, CLR, CTR and RAM1-3 mRNA were used as a reference to quantify the relative amount of mRNA in the sample, using the formula X_0_/R_0=_ 2^CtR−CtX^. Where X0 is the amount of target mRNA, R_0_ is the amount of housekeeping gene mRNA, C_t_R is the C_t_ value of the housekeeping gene and C_t_X is the C_t_ value of the target.

All statistical analyses were performed using Graph Pad Prism 9. Statistical significance for RT-qPCR and immunohistochemistry were determined using Mann-Whitney, Student’s t-test. Data were expressed as mean ± standard error of the mean (SEM), *n* refers to the number of rats, *P < 0.05, was considered significant.

## Results

### Gene expression of CGRP family of peptides in TG of male and female rat

**Fig. 1:**
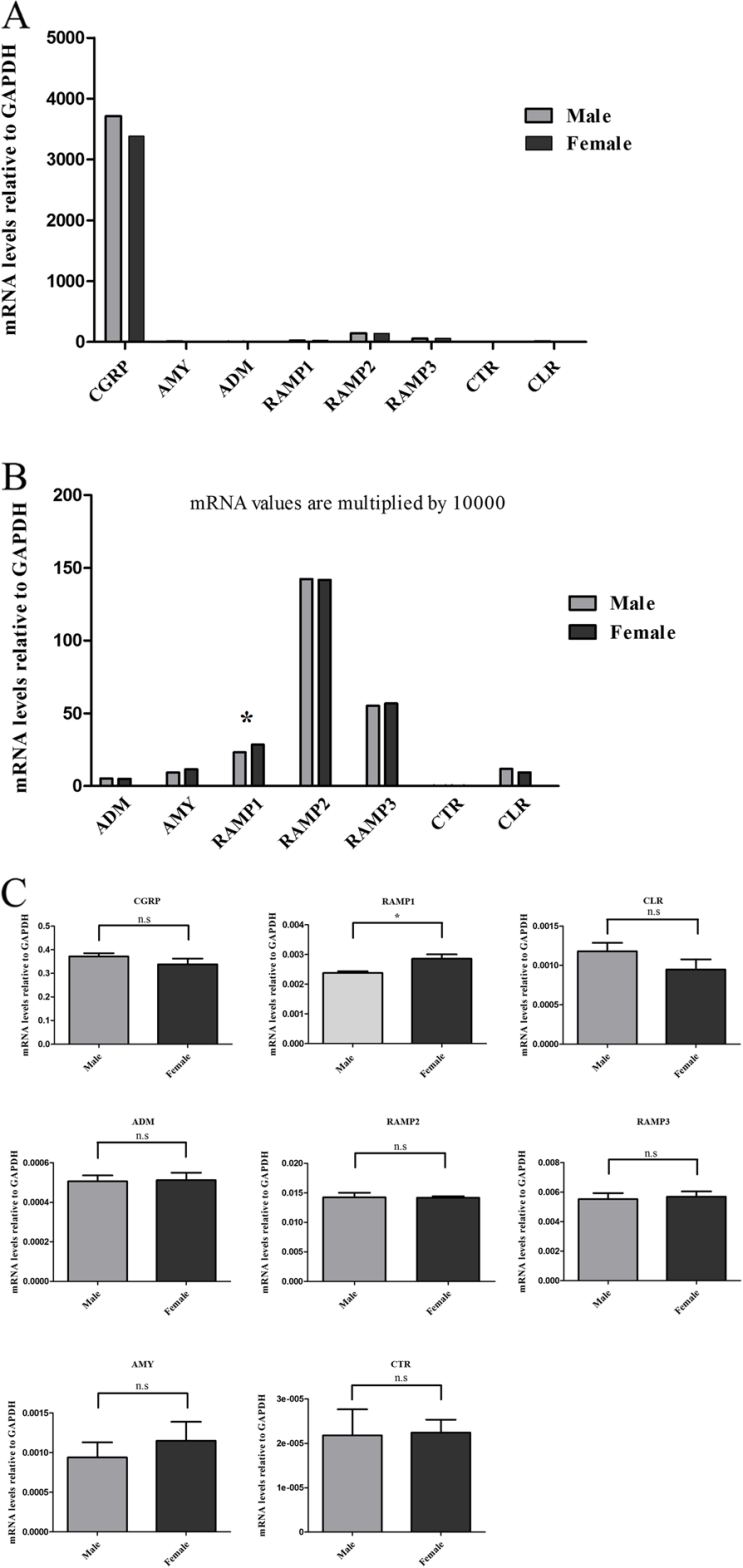
Gene expression of CGRP family of peptides in female and male TG. **A**. CGRP mRNA level expression in TG was significantly higher in comparison to other genes. **B** There were expressions of all genes in male and female TG except for CTR. **C** RAMP1 mRNA levels were significantly higher in TG of female compared to male rats. Expressions were normalized to housekeeping gene GAPDH, *n* = 6. Data were obtained by RT-qPCR and are expressed as mean ± S.E.M, *P < 0.05

The gene expression analysis was performed to understand the expression levels of CGRP, ADM, AMY, RAMP1, RAMP2, RAMP3, CTR and CLR in the TG tissue of male and female rats. The results shown in Fig. 1 revealed several key findings.

Firstly, the expression of CGRP mRNA in TG was considerably higher compared to the other genes analyzed. There was no significant difference between male and female TG in expression of the CGRP mRNA (Fig. 1A). Conversely, the mRNA expression levels of other peptides and receptors in TG were minimal, almost negligible, in comparison to CGRP mRNA (Fig. 1A). Secondly, expression was detected for all genes in both male and female TG, except for CTR (Fig. 1B), as indicated by the Ct values provided in supplemental Table 1. Thirdly, differences in mRNA expression levels between male and female rats were observed only for CGRP, AMY, CLR and RAMP1, genes (Fig. 1C). While CGRP and CLR mRNA levels tended to be higher in male TG compared to female TG, these differences were not statistically significant. Conversely, AMY and RAMP1 mRNA levels were higher in females. Notably, RAMP1 mRNA levels were significantly higher in female TG compared to male TG (Fig. 1C).

### Immunohistochemistry

The distribution of CGRP expression has been performed in rat TG, and was here related to previous studies [24, 30]. We here confirm these results and observed that the CGRP immunoreactivity was detected mostly in the cytoplasm in a granular-like pattern of small to medium sized neurons, and in thin unmyelinated fibers (C-fibers). Further, there was no significant difference in the number of CGRP positive immunoreactive cells in the TG between male (48 ± 1.6%) and female (42 ± 3%) rats [24].

The distribution of AMY, ADM and CT, and their receptors have previously only been demonstrated in the TGs from young male rat [30]. In the present study we examined in parallel possible differences in expression of the CGRP family of neuropeptides: CT, AMY, and ADM, and their receptors in male and female rat TGs, using indirect immunofluorescence staining for detection and localization of these proteins.

### Distribustion of CGRP family of neuropeptides and their receptors in TG of male and female rat

#### Peptide expression and distribution

Because the antibody utilized before [30] was not selective for CT and appears to be raised against CGRP [15], two other antibodies were used in this study in order to detect possible expression of CT (for details, see Table 1).

Results with either of these antibodies demonstrated that there was no positive staining of the CT protein in the TG or in the dura mater. This indicates that there is no CT expression in the TG neurons, satellite glial cells (SGCs), or located in the nerve fibers.

To validate the expression of amylin in the TG and dura mater, we employed three antibodies from three different companies to detect amylin protein in the TG (for details, see Table 1).

The findings indicated that the antibody from Santa Cruz (SC-377530) demonstrated no AMY immunoreactivity in the TG. In contrast, LS-C352341 antibody from Life Span Bioscience showed abundant immunoreactivity in TG. This did not provide clarification whether staining detected AMY or exhibited non-specific binding and false positive. The antibody from ThermoFisher Scientific (PA5-32261) demonstrated a very weak expression of AMY in a few TG neurons. However, this suggested cross-reactivity of this antibody with CGRP as reported recently [33, 34]. Collectively, the work suggests that there is no or very low AMY expression in the rat TG. This mirrors the results from mRNA analysis in Fig. 1.

**Fig. 2:**
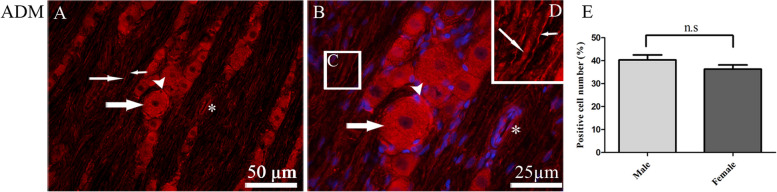
Immunohistochemistry of ADM expression in TG. **A** and **B** ADM immunoreactivity was found in the cytoplasm of TG neurons (thick arrows), in the cytoplasm of SGCs (arrowheads), in the thick fibers (Aδ-fibers) (thin arrows), and in the blood vessel walls (asterisk markers). **D** In addition, ADM immunoreactivity was found in the cytoplasm of Schwann cells surrounding fibers (short thin arrows). **D** Higher magnification of image. **C** Blue color represents nucleus staining with DAPI. **E** The bar graph shows 36 ± 1.8% of the total number of neurons in females and 40 ± 2.1% in males expressed ADM immunoreactivity in TG. Data are presented as the mean ± S.E.M and *n* = 6. A *p*-value < 0.05 was considered significant

ADM immunoreactivity was observed in the cytoplasm of TG neurons and satellite glial cells (SGCs). Additionally, a weak expression was observed in Aδ-fibers (Fig. 2A-C). ADM immunoreactivity was also detected in Schwann cells surrounding the fibers (Fig. 2C and D).

**Fig. 3:**
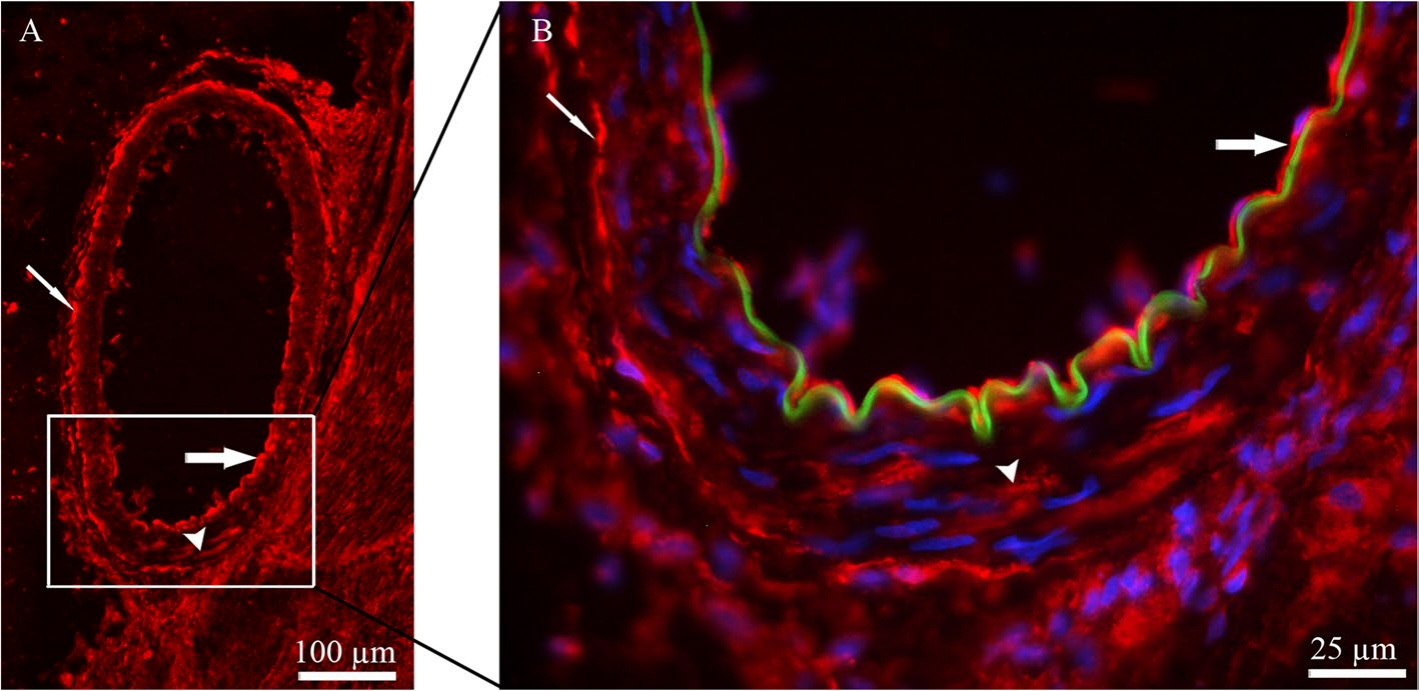
Immunohistochemistry of ADM expression in the blood vessel wall. **A** and **B**. ADM was expressed mainly in vascular endothelium (thick arrows) and in adventitia layer (thin arrows). In addition, a few cells expressed ADM in the smooth muscle cell layer (arrowheads). Green color in image B, represents internal elastic lamina, and blue color represents nucleus staining with DAPI

Furthermore, ADM expression was observed in vessel walls of basilar artery, primarily localized in the vascular endothelium, with some expression in the smooth muscle cell layer (SMCs) (Fig. 3). The percentage of ADM-expressing TG neurons was found to be 36 ± 1.8% in females and 40 ± 2.1% in males, with no significant difference between the sexes (*p* > 0.05) (Fig. 2E).

#### Receptor expresssion and distribution

**Fig. 4:**
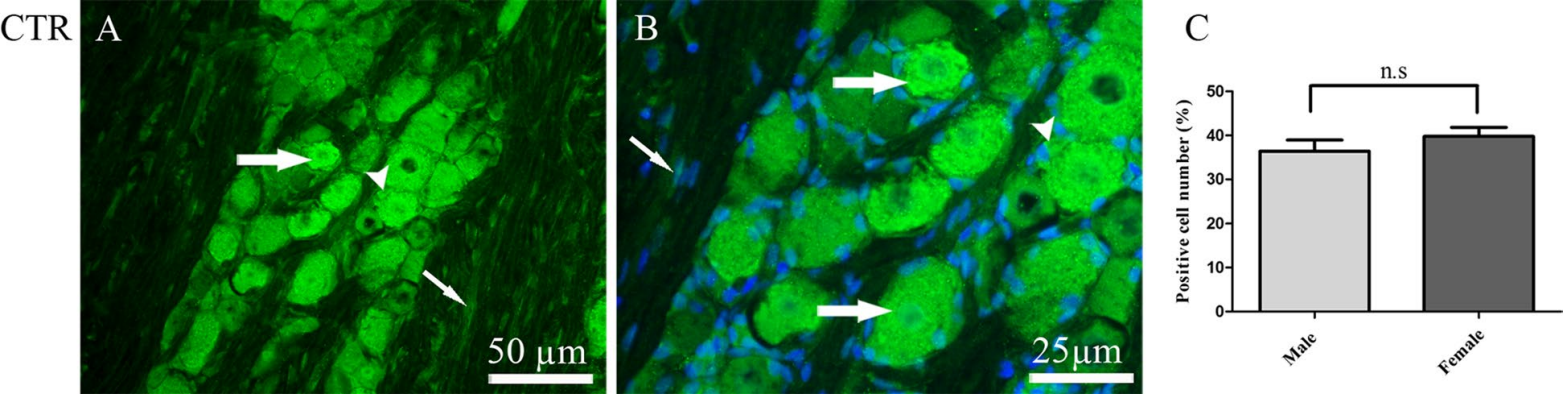
Immunohistochemistry of CTR expression in TG. **A** and **B** CTR immunoreactivity was found in both cytoplasm and the nucleus of neurons (thick arrows), and in the cytoplasm of SGCs (arrow heads). In addition, a weak CTR immunoreactivity was seen in the nuclei of the Schwann cells surrounding the Aδ-fibers (thin arrows). Blue color represents nucleus staining with DAPI. **C** The bar graph shows that the number of CTR immunoreactivity cells was higher in females (39.7 ± 2%) compared to males (36.3 ± 2.5%). However, this was not significant. Data are presented as the mean ± S.E.M and *n* = 6. A *p*-value < 0.05 was considered significants

Using the available and specific CTR antibody showed CTR expression in both cytoplasm and nucleus of all sized neurons, and in the cytoplasm of SGCs. In addition, CTR immunoreactivity was found in the nuclei of the Schwann cells (Fig. 4A and B). There were no differences between the number of CTR immunoreactivity cells in females (39.7 ± 2%) and in males (36.5 ± 2.5%) (Fig. 4C).

**Fig. 5:**
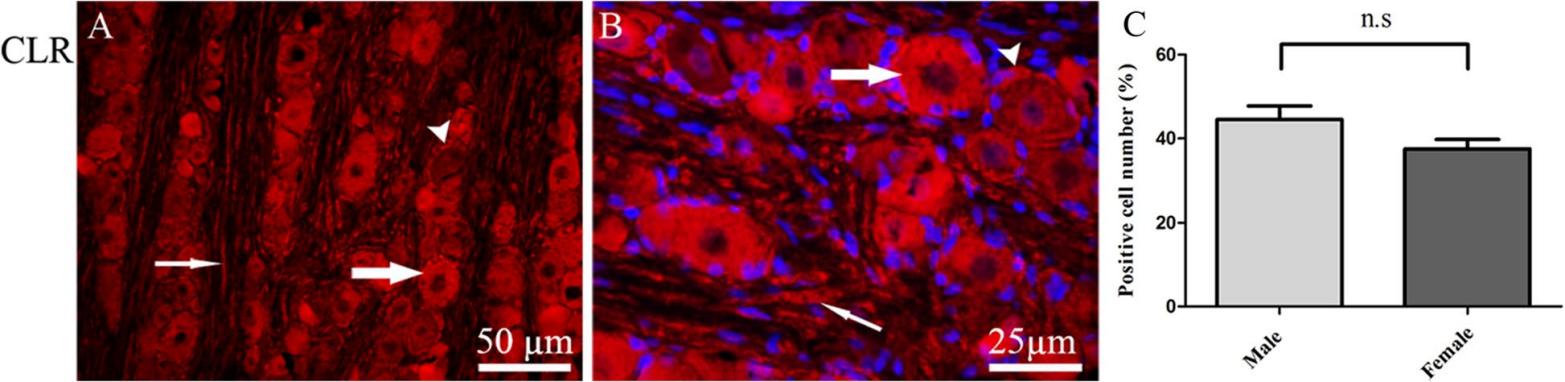
Immunohistochemistry of CLR expression in TG.** A** and **B** CLR immunoreactivity was seen in the cytoplasm of neurons (thick arrows), in the cytoplasm of SGCs (arrowheads) and in Aδ-fibers (thin arrows). **C** The bar graph shows 44.5 ± 3.2% in males and 37 ± 2.5% in females of the total number of neurons, expressed CLR immunoreactivity in TG. Data are presented as the mean ± S.E.M and *n* = 6. A *p*-value < 0.05 was considered significant

CLR was expressed in the cytoplasm of medium to large sized neurons. Further, CLR immunoreactivity was observed in the cytoplasm of SGCs and in Aδ-fibers (Fig. 5A and B). Cell counting demonstrated that the number of CLR positive neurons was higher in male TG (45.2 ± 3.2%) compared to female TG (38 ± 2.5%), however, this was not significant (*P* > 0, 05) (Fig. 5C).

In the present study as well as in our earlier studies we confirm that RAMP1 was expressed in the cytoplasm of large to medium sized neurons and in the Aδ-fibers. In addition, the number of RAMP1 positive neurons was significantly higher in females (41 ± 2.7%) as compared to males (22.5 ± 2.1%; *p < 0, 05) [24].

**Fig. 6:**
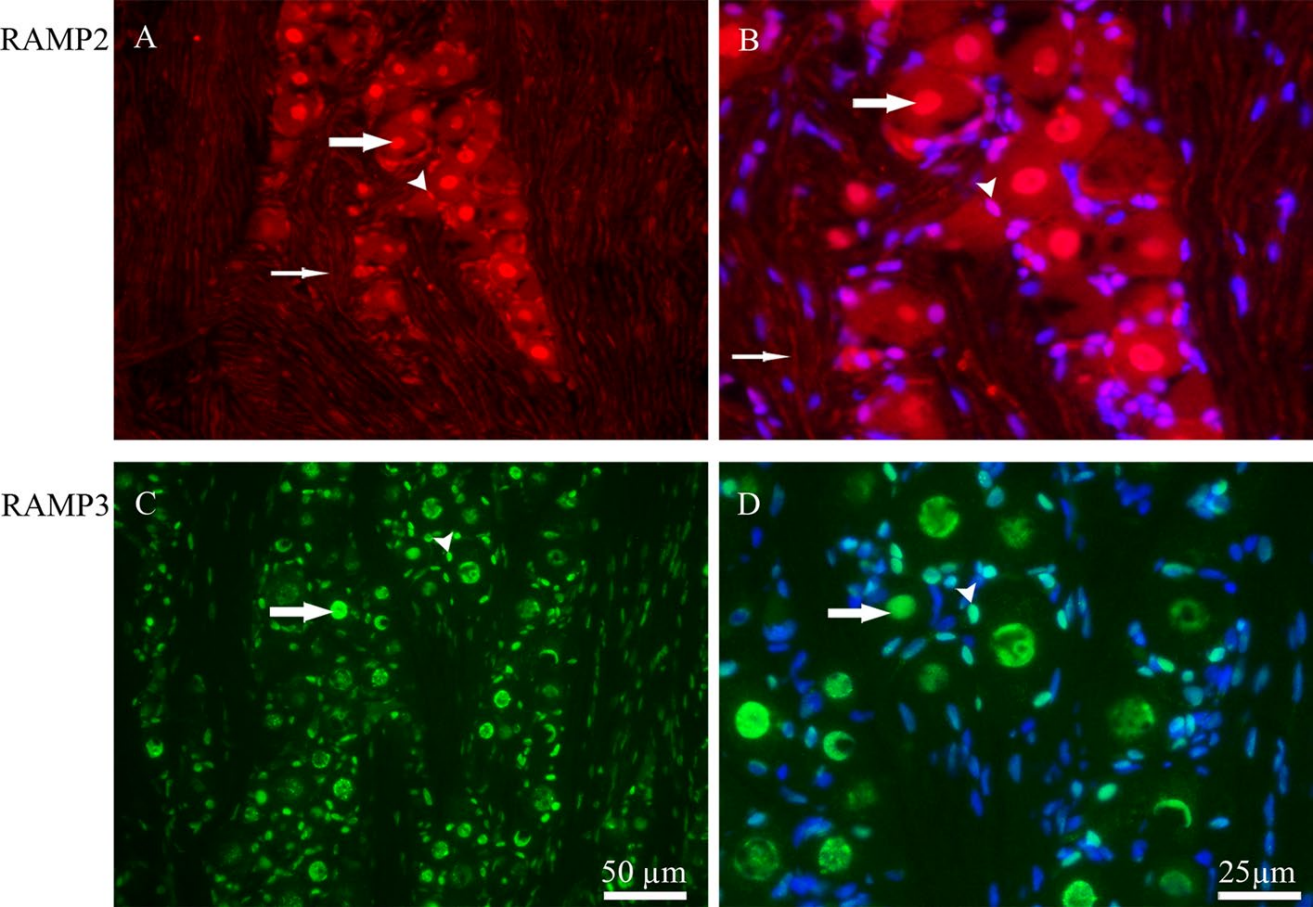
Immunohistochemistry of RAMP2 and RAMP3 expression in TG. **A** and **B** RAMP2 immunoreactivity was located in nuclei of neurons (thick arrows) and SGCs (arrowheads). In addition, a weaker expression of RAMP2 was observed in Aδ-fibers (thin arrows). **C** and **D** RAMP3 immunoreactivity was seen in the nucleus of neuron (thick arrows) and SGCs (arrowheads). Blue colors represent nucleus staining with DAPI

The distribution of the receptor components RAMP2 and RAMP3 was examined in detail. RAMP2 immunoreactivity was found in the nucleus of TG neurons and in the SGCs. In addition, a weak expression of RAMP2 was found in Aδ-fibers (Fig. 6A and B). RAMP3 was expressed in the nucleus, of both TG neurons and SGCs. Further, RAMP3 was expressed in the nucleus of Schwann cells (Fig. 6C and D). There was no difference in the RAMP2 and RAMP3 proteins expression between male and female rats. Since these proteins are mainly expressed in large number in the nuclei of neurons and glial cells, no cell counting could therefore be done.

#### Axonal localiszations of peptides and receptors in TG and Dura mater

**Fig. 7:**
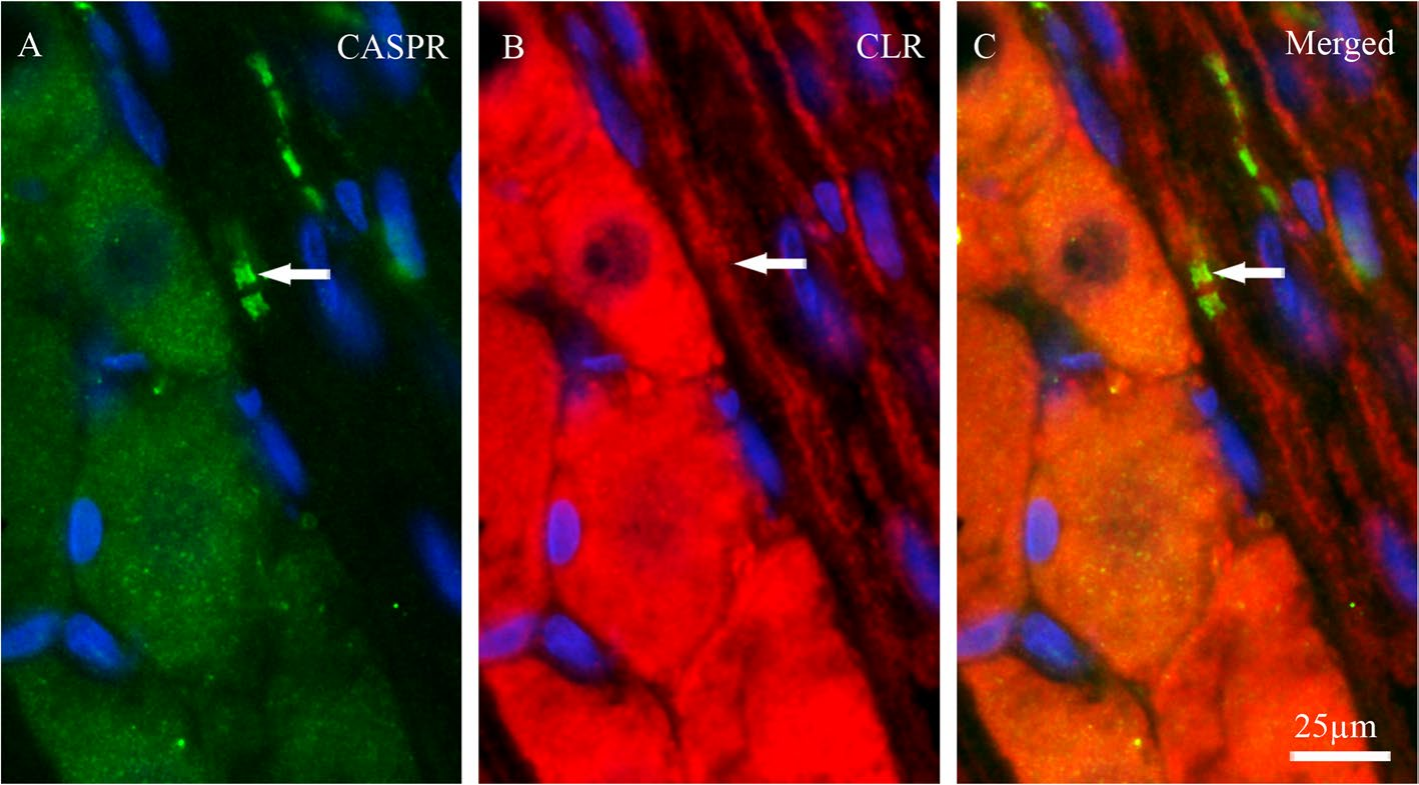
Co-localization of CLR with CASPR. **A** CASPR immunoreactivity. **B** CLR immunoreactivity. **C** Merged. Double staining of CLR with CASPR showed that CLR co-localized with CASPR in the paranodal regions flanking the nodes of Ranvier (arrows). Blue colors represent nucleus staining with DAPI

**Fig. 8:**
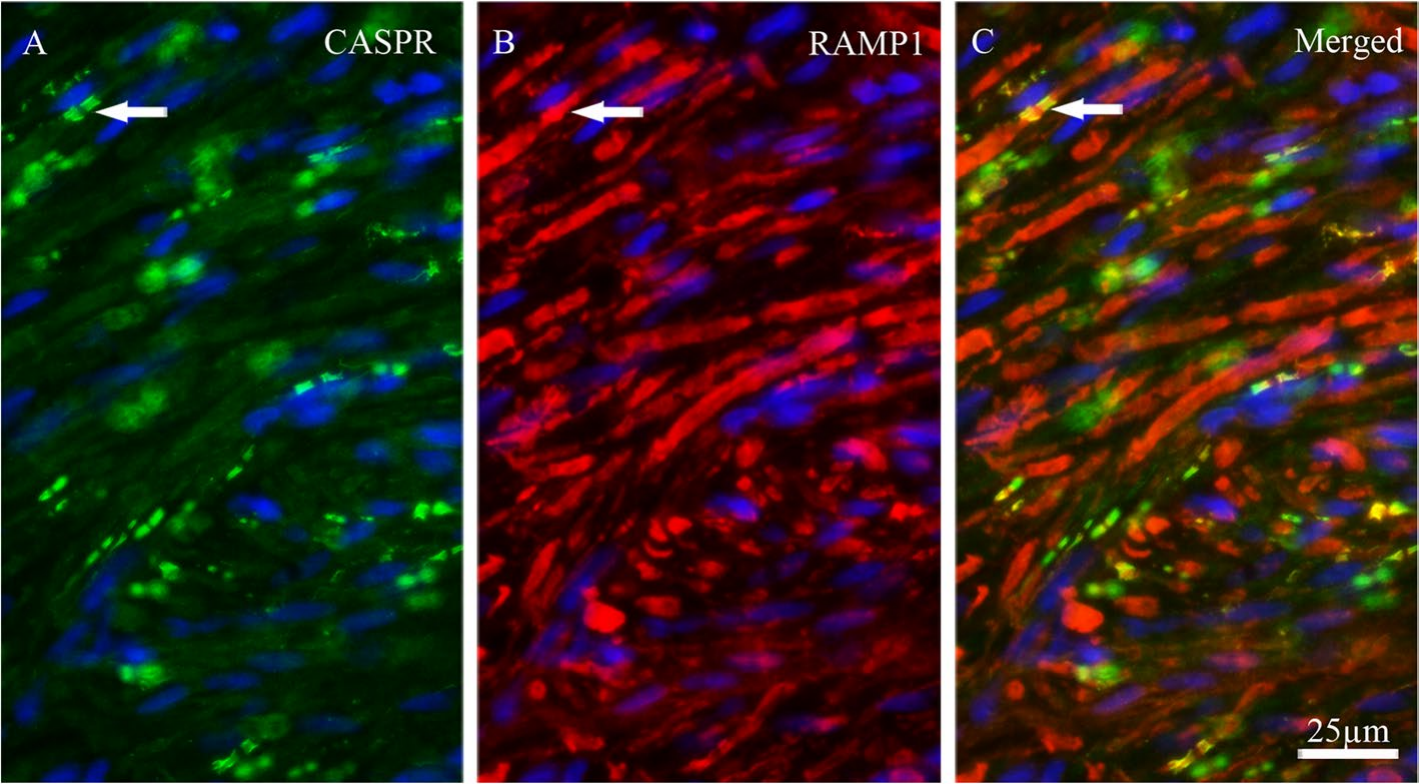
Co-localization of RAMP1 with CASPR. **A**. CASPR immunoreactivity. **B** RAMP1 immunoreactivity. **C** Merged. Double staining of RAMP1 with CASPR showed that RAMP1 co-localized with CASPR in the paranodal regions flanking the nodes of Ranvier (arrows). Blue colors represent nucleus staining with DAPI

**Fig. 9:**
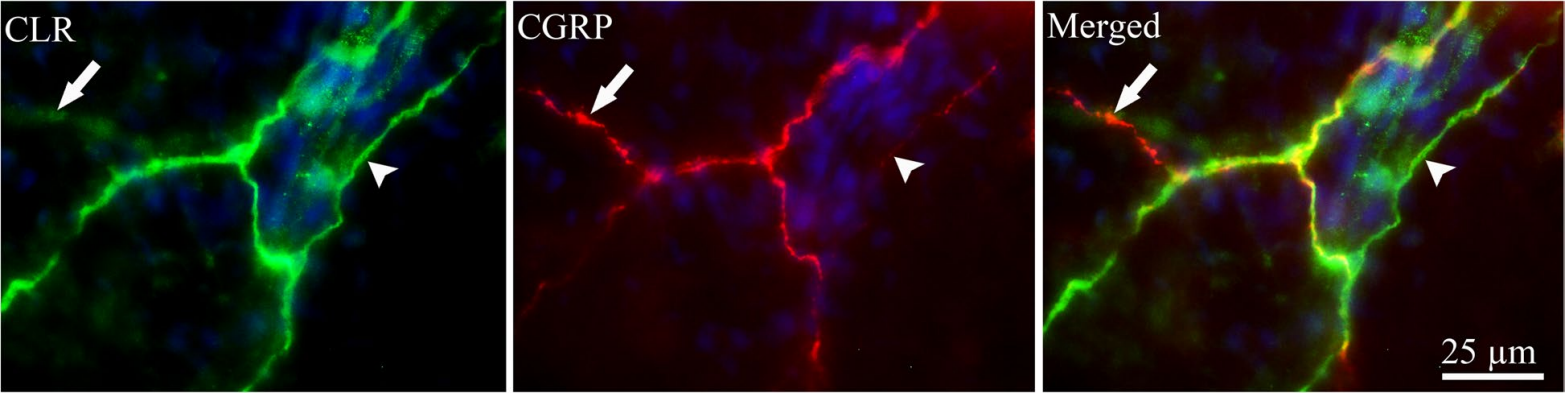
CLR and CGRP immunoreactivity in rat dura mater. Double staining of CLR with CGRP revealed bundles of C- and Aδ-fibers projecting intimately in the rat dura mater. C-fibers displayed immunoreactivity for CGRP (arrow), while Aδ-fibers were immunoreactive for CLR (arrowhead)

Double immunohistochemistry was performed to investigate potential co-localization of ADM, AMY, CTR, CLR, RAMP1, RAMP2 and RAMP3 with contactin associated protein 1 (CASPR). CASPR is a transmembrane protein expressed at the paranodal regions flanking the nodes of Ranvier of myelinated fibers [31]. The results showed that only the CGRP receptor elements; CLR and RAMP1 co-localized with CASPR in paranodes (Figs. 7 and 8, respectively). In addition, there was expression of CLR in Schwann cells around the nodes. There was no co-localization for ADM, AMY, CTR, RAMP2 and RAMP3 with CASPR in the paranodal region in TG tissue.To further investigate the CGRP family of receptor components expression, the same double staining with CASPR and CGRP were performed in rat dura mater spreads. Only CLR and RAMP1 were observed to be expressed in dural Aδ-fibers, confirmed by CASPR expression in the same fibers. Double staining for CLR and CGRP revealed closely intertwined bundles of C- and Aδ-fibers projecting throughout the rat dura mater (Fig. 9).

No immunoreactivity for ADM, AMY or their receptors was observed in the rat dura mater.

## Discussion

The first demonstration of CGRP in the TVS was presented in 1984 which marked a turning point in migraine research [35, 36]. It has stood the test of time and brought attention to CGRP and its receptor, leading to the development of successful migraine therapies, including monoclonal antibodies and gepants [37–39]. Subsequent work has paid attention to the other members of the CGRP family of peptides and receptors with the hope of helping patients with poor responses to the currently available CGRP medications. In the present study we first examined quantitative gene expression of mRNA of the CGRP family of peptides (CGRP, ADM, AMY, CT) and receptors (CLR, CTR, RAPM1-3) in the TG, followed by protein analysis.

### Ligands expression analysis

As shown in Fig. 1, the CGRP mRNA was several folds more prevalent as compared to the mRNA of other peptides such as ADM, AMY and CT. This suggests that CGRP has a predominant role over the other family members in the TVS, being present in about 40–50% of the TG neurons in both male and female rats, and in humans [12, 24].

The mRNA expressions of ADM, AMY and CT were relatively minor in the TG (Fig. 1) which suggests that their role would at best be minor. Rees and Hendrix et al.. have developed several antibodies which were verified to be more specific as compared to commercially available antibodies, showing that the immunoreactivity of CT and AMY were indeed very low [15, 40], agreeing with our mRNA analysis.

In a recent report AMY was proposed to be expressed in small to medium sized rat TG neurons [30]. However, this suggested a cross-reactivity of the applied antibody with CGRP as recently reported [34]. Therefore, in the present work we used a set of more specific antibodies, but did not identify a clear and specific expression of AMY in rat TG. In addition, Rees et al.. noted a rich expression of AMY immunoreactivity in upper dorsal root ganglia but no AMY expression in the TG [33]. Thus, collectively the work suggests that AMY and CT, released from the pancreas and thyroid, respectively, might reach the TVS via the circulation. However, the levels of these hormones in the blood circulation are reported to be relatively minor [41, 42]. On the other hand, Ashina and colleagues recently reported that a monoclonal antibody targeting PACAP has demonstrated efficacy in migraine prophylaxis [43], despite PACAP being less expressed in the TG compared to CGRP. Therefore, the low expression of AMY and CT in the TG should not be a reason to exclude their potential involvement in pathologies of the TVS. More research and experimentation are needed to fully understand their roles.

ADM has been reported to be expressed in endothelium [44]. The present results confirmed that ADM was mainly expressed in the endothelium with a weaker expression in SMCs of the vessels walls (Fig. 3). In the present study mRNA analysis showed relatively low levels of the ADM mRNA in the TG (Fig. 1). Thus, positive ADM immunoreactivity was verified mainly in vascular endothelium. Although, some positive staining was also observed in TG neurons and satellite glial cells (SGCs). However, the immunoreactivity for ADM was much weaker when compared to CGRP immunoreactivity. The weaker ADM immunoreactivity in TG neurons and SGCs, together with the low mRNA level, suggests that ADM may play a limited role in trigeminal signaling pathways compared to CGRP.

### Receptor expression analysis

Results from the present study together with previous work have demonstrated that the CGRP receptor was observed in a large portion of TG neurons and satellite glial cells [30]. In contrast, ADM receptors (CLR with RAMP2 or RAMP3) were not observed in the blood vessel walls or in other parts of the TVS except for the TG.

Interestingly, CTR by itself and the AMY1 receptor (CTR/RAMP1) were found to a high degree within the TG. Since we did not find CT immunoreactivity in the TVS, the CTR based receptors (CTR and AMY1R) would appear to lack CT as an activator within the TVS. It is likely they can be stimulated either by the circulating hormones AMY and CT, or by the abundance of CGRP within the TVS (Fig. 1). Interestingly, functional studies on vasodilation of human intracranial arteries revealed that the responses to CT and AMY were small and about 2 log units less in potency [45, 46]. Since the circulating levels of these hormones are low, they may only give weak vasodilator effects at high concentrations. Together we may question the role of these receptors at least as vasodilators. However, there was no expression of CTR or AMY1 receptors or their ligands in the nerve fibers of rat dura mater which indicates a limited role in that context.

We recognize that the receptor expression patterns observed in rats may not fully reflect those in humans. To address this limitation, we emphasize the need for further investigations in human tissues. Therefore, we plan to conduct a similar study focusing on human middle meningeal tissues, specifically the middle meningeal artery (MMA) and dura mater, to validate the relevance of our findings to human migraine pathophysiology.

### Sex differences

Despite the high prevalence and enormous burden of migraine among women, there are few studies on differences in CGRP expression and the role of gender in the CGRP family of peptides and their receptors [8, 47]. Recently, we counted the number of CGRP expressing neurons and found that 48% of the total number of neurons in males and 42% in females expressed CGRP immunoreactivity in TG [24] which is in agreement with an earlier study on rat and human TG [12]. We also reported that the CGRP receptor component (RAMP1) was expressed in the cytoplasm of medium to large size neurons and in Aδ-fibers, also the number of positive neurons was significantly higher in females (41%) compared to males (22%) [24]. The elevated expression of RAMP1 in female TG may indicate a potential involvement of RAMP1 in migraine susceptibility or pathogenesis in females.

In addition, the differential efficacy of gepants between men and women may be influenced by higher RAMP1 expression in the TG of females. RAMP1 modulates CGRP receptor activity, potentially making women more responsive to CGRP antagonists like gepants. While gepants are effective in men, their efficacy may be somewhat reduced, as reported in a recent study [48]. This potential sex-based difference in treatment response underscores the need for further research into the molecular mechanisms underlying migraine therapies.

### Examination of CGRP family ligands and receptors in rat dura mater

In agreement with previous studies [9, 12], CLR imunoreactivity was located in the cytoplasm of both neurons and SGCs, as well as in Aδ-fibers (Fig. 5A and B). Interestingly, only RAMP1 and CLR (CGRP canonical receptors) were co-localized with CASPR in the paranodal regions of the Aδ-fibers (Figs. 7 and 8). This was in agreement with a previous study for RAMP1 [31]. The co-localization of CLR and RAMP1 on the Aδ-fibers with CASPR suggest a potential role for these receptors in modulating trigeminal nerve function and neuronal excitability, which could have important implications for understanding migraine pathophysiology. Additionally, the close association of C- and Aδ-fiber bundles stained for CLR and CGRP suggests a significant role for CGRP in these fibers. Curiously, we observed no immunoreactivity for CTR, RAMP2 or RAMP3, in the rat dural Aδ-fibers. The same was true for ADM and AMY in rat dural C-fibers. This suggests that CGRP alone, in the CGRP family of peptides, is situated to activate its receptor on parallell Aδ-fibers via extrasynaptic signalling in the rat dura mater. These results highlight the importance of CGRP as a neuromodulator in the TVS.

We acknowledge the limited translatability of findings from animal to human physiology, particularly regarding CGRP family ligands and receptors in the dura mater. However, a future study is planned to directly investigate these mechanisms in human tissues, which will help bridge this gap in the current study.

## Conclusion

This study provides valuable insights into the distribution of the CGRP family of peptides and their receptors in the TVS. CGRP mRNA levels in the TG were significantly higher than those of other genes. Additionally, the co-localization of CLR and RAMP1 on Aδ-fibers with CASPR suggests a potential role for these receptors in modulating trigeminal nerve function and neuronal excitability, with implications for migraine pathophysiology. Furthermore, RAMP1 mRNA levels were significantly higher in female TG compared to males, indicating sex-specific differences in gene expression. These findings underscore key role of CGRP and its receptor in the functional significance of the sex-related variations.

## Supplementary Information

Supplementary Material 1.

## Data Availability

No datasets were generated or analysed during the current study.

## Abbreviations

ADM: Adrenomedullin
AMY: Amylin
BSA: Bovine serum albumin
CT: Calcitonin
CGRP: Calcitonin gene-related peptide
CTR: Calcitonin receptor
CLR: Calcitonin -like receptor
cDNA: Complementary DNA, DAPI: 4', 6-diamidino-2-phenylindole
DNA: Deoxyribonucleic acid
GAPDH: Glyceraldehyde-3-phosphate dehydrogenase
mRNA: Messenger RNA
PBS: Phosphate buffer saline
PBS-T: Phosphate buffer saline-Triton X
PF: Paraformaldehyde
RNA: Ribonucleic acid
RT-qPCR: Real-time quantitative Polymerase Chain Reaction
SGC: Satellite glial cell
TG: Trigeminal ganglion
TVS: Trigeminovascular system
RAMP1: Receptor activity-modifying protein 1
RAMP2: Receptor activity-modifying protein 2
RAMP3: Receptor activity-modifying protein 3
