# Supplementary material for: Sex differences in expression of CGRP family of receptors and ligands in the rat trigeminal system
Source: J Headache Pain. 2024 Nov 8;25(1):193. doi: 10.1186/s10194-024-01893-1 (PMC11545840; doi:10.1186/s10194-024-01893-1)
Supplement: Supplementary file 1 — Supplementary Material 1. [file 10194_2024_1893_MOESM1_ESM.docx]

**Supplemental table 1. CT values of CGRP, AMY, ADM, RAMP1, RAMP2, RAMP3, CLR and CTR genes from RT-qPCR experiment in rat TG**.

| CT value | | | |
| --- | --- | --- | --- |
| **Gene Name** | **Male**  **(Mean ± SEM)** | **Female**  **(Mean ± SEM)** | **N** |
| CGRP | 19.5 ± 0.07 | 21.5 ± 0.1 | 6 |
| AMY | 29.6 ± 0.4 | 28.1± 0.4 | 6 |
| ADM | 29.0 ± 0.1 | 30.1 ± 0.1 | 6 |
| RAMP1 | 28.8 ±0.1 | 26.1 ± 0.1 | 6 |
| RAMP2 | 24.0 ± 0.05 | 25.9 ± 0.1 | 6 |
| RAMP3 | 26.4 ± 0.04 | 27.2 ± 0.1 | 6 |
| CLR | 27.7 ± 0.1 | 30.1 ± 0.1 | 6 |
| CTR | 36.6 ± 0.3 | 34.8 ± 0.4 | 6 |
